# Expression of Concern: Long non-coding RNA PSMA3-AS1 enhances cell proliferation, migration and invasion by regulating miR-302a-3p/RAB22A in glioma

**DOI:** 10.1042/BSR-2019-1571_EOC

**Published:** 2023-01-19

**Keywords:** cell proliferation, glioma, miR-302a-3p, PSMA-AS1, RAB22A

The Editorial Office has been made aware of potential issues surrounding the scientific validity of this paper, hence has issued an expression of concern to notify readers whilst the Editorial Office investigates. Some of the western blots seem to appear in other papers by unrelated authors. The Figure 6E RAB22A and B-actin blots seem to appear as the Figure 5B RAB14 and GAPDH bands from Xu et al. 2020 (https://doi.org/10.1186/s13046-020-01556-4). Vertically compressed, elongated versions the Figure 3C western blots also seem to appear as the Figure 3A blots from Feng et al. 2019 (https://doi.org/10.1186/s13046-019-1070-x).

